# Feasibility of a combined aerobic and cognitive training intervention on cognitive function in cancer survivors: a pilot investigation

**DOI:** 10.1186/s40814-018-0242-3

**Published:** 2018-02-17

**Authors:** Brent M. Peterson, Cynthia Johnson, Kaylene R. Case, Daniel Y. K. Shackelford, Jessica M. Brown, Trent L. Lalonde, Reid Hayward

**Affiliations:** 1 Department of Kinesiology and Health Science, Biola University, 13800 Biola Ave., La Mirada, CA 90639 USA; 2 Department of Exercise Science, Carroll University, 100 N. East Ave., Waukesha, WI 53186 USA; 3 School of Sport and Exercise Science, University of Northern Colorado Cancer Rehabilitation Institute, University of Northern Colorado, 501 W. 20th St., Greeley, CO 80639 USA; 4 School of Psychological Sciences, University of Northern Colorado, Greeley, CO USA; 5 Department of Applied Statistics and Research Methods, University of Northern Colorado, Greeley, CO USA

**Keywords:** Exercise, Cognitive function, Chemotherapy-related cognitive impairment, Cancer-related cognitive impairment, Cancer

## Abstract

**Background:**

Cancer-related cognitive impairment (CRCI) may negatively affect upwards of 75% of cancer patients. Exercise and cognitive training, independently, may increase functional capacity and aspects of cognitive function. Yet, combined training protocols have not been evaluated in cancer survivor populations. Therefore, the aim of this study was to explore the feasibility of a quasi-randomized, controlled, exploratory, repeated-measures aerobic and cognitive training intervention on cognitive function in participants undergoing treatment for cancer (*N* = 28).

**Methods:**

Pre- and post-physical and cognitive assessments were administered. A 36-session (approximately 12 weeks) computer-based cognitive (COG), aerobic (AER), cognitive and aerobic (AER + COG), and flexibility (CON) training intervention was completed. Dependent measures *t* tests and pre- to post percentages were then calculated to address within-group changes for each dependent variable.

**Results:**

Within-group measures revealed that the AER logical memory scores (pre- to post mean difference [2.3], 95.0% CI [0.9, 3.7], percentage change [32.7%]), delayed recall scores (pre- to post mean difference [2.1], 95.0% CI [0.3, 3.9], percentage change [27.2%]), block design scores (pre- to post mean difference [1.7], 95.0% CI [0.2, 3.2], percentage change [19.0%]), and letter-number sequencing scores (pre- to post mean difference [1.0], 95.0% CI [0.2, 1.8], percentage change [12.3%]) all increased. Aspects of verbal fluidity scores increased in the CON group. However, all cognitive scores (AER + COG and COG groups) failed to increase.

**Conclusions:**

Aerobic training for CRCI may positively impact cognitive function. Individually, these methods may appropriately address CRCI, but combined training of this nature may be too demanding for patients undergoing treatment for cancer. However, larger randomized trials are needed to substantiate this protocol in large-scale cancer rehabilitation centers.

## Background

Cancer patients experience an array of negative side effects during and following chemotherapy. Up to 75% of cancer patients undergoing treatment experience some degree of cognitive dysfunction [1, 2], and up to 60% experience a deterioration in cognitive function even after chemotherapeutic treatments have completed [3]. This phenomenon has been described as chemotherapy-related or cancer-related cognitive impairment (CRCI) [4, 5], and it can have far-reaching effects on reaction time, organizational skills, linguistic abilities, attention [6], quality of life, activities of daily living, memory, and concentration [7–9].

While the exact mechanisms responsible for CRCI are unclear, there appear to be primary and secondary mechanisms, and the syndrome itself is likely to occur through multiple pathways. Specific pathways affected may be dependent on the type of chemotherapy (e.g., alkylating agents, antimetabolites, anti-tumor antibiotics) and may even be the result of biological processes associated with the cancer itself [10–14]. Selected studies suggest that CRCI could be related to host characteristics such as age, socioeconomic status, ethnicity, disease stage, or body mass index [6]. Several studies have suggested that CRCI is the product of a reduction in white matter integrity and impaired brain activation. Cancer survivors also have elevated levels of circulating cytokines which supports the possibility that immune function dysregulation accompanied by systemic inflammation may be a primary means by which CRCI is mediated [15–17].

Treatment and management of CRCI has proven to be a difficult task due to the fact that it has yet to be fully characterized. However, studies have shown that exercise or cognitive training may mitigate some components of CRCI [18–23]. Ferguson et al. [24] reported that cancer survivors completing Memory and Attention Adaptation Training showed significant improvements in verbal memory. Von Ah et al. [25] showed that breast cancer survivors completing one of two modified versions of the Advanced Cognitive Training for Independent and Vital Elderly (ACTIVE) program improved memory performance, speed of processing, perceived cognitive functioning, symptom distress, and quality of life. Although exercise and physical activity interventions have been preliminary in nature up to this point, they have been shown to alleviate CRCI. In a sample of over 400 cancer patients, those who self-reported higher levels of physical activity also reported less memory loss [26]. Interventions such as tai chi and Qigong have also been shown to improve both objective and subjective measures of cognitive function in cancer survivors [27–29]. These clinical studies have been supported by animal studies showing that exercise improves spatial memory and object recognition in rodents receiving chemotherapy [30, 31]. While aerobic exercise alone or cognitive training alone have been shown to increase quality of life and cognitive function in apparently healthy adults as well as cancer survivors [32–39], it is unclear whether simultaneously combining these two interventions could provide additive or synergistic benefits on cognitive function in cancer survivors. Thus, the purpose of this study was to determine if the combination of aerobic exercise and cognitive exercise could improve cognitive function when compared to either intervention alone.

### Objectives

1. To design, implement, and evaluate methodology aimed at reducing CRCI in patients undergoing cancer treatment for safety, efficacy, and participant adherence.
2. To pilot and explore safety concerns, efficacy of methodology, and participant adherence for the purposes of informing investigators of shortcomings and potential hurdles to address.
3. To examine and test clinically relevant forms of exercise that may provide cost-effective attenuation of CRCI symptoms for the purposes of informing large-scale multi-site cancer rehabilitation programs intent on addressing CRCI.

## Methods

### Trial design

**Fig. 1:**
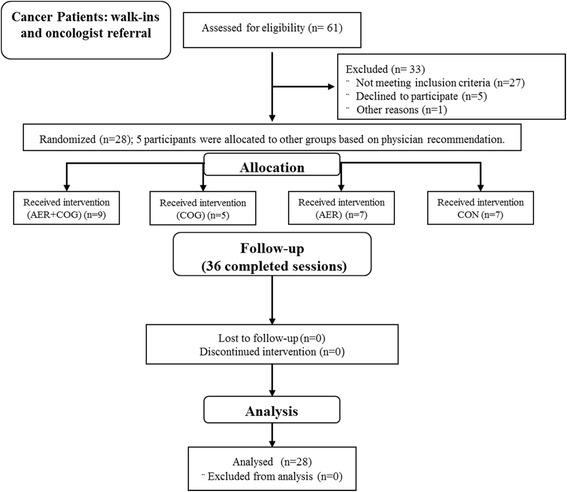
Experimental design. AER + COG aerobic and cognitive training, COG cognitive training, AER aerobic training, CON flexibility training only

This pilot study consisted of a 36-session intervention (approximately 12.0 weeks) that included aerobic exercise training and computer-based cognitive training (Fig. 1). Subjects were randomly assigned to receive aerobic training alone (AER), cognitive training alone (COG), or a combination of aerobic and cognitive training (AER + COG). A cancer control group (CON) participated in 36.0 sessions of a 30-min flexibility only intervention. Directly following the completion of 30 min of AER, COG, or AER + COG training (while seated on the Motion Fitness Brain Bike^®^), participants then completed the 30-min flexibility training intervention for a total of an hour of training. This was to ensure that any changes in cognitive function were not the result of personal interactions that occurred between subjects and those individuals supervising the intervention. A summary of the group stratification, training schedule, and cognitive training exercises is in Table 1. All aerobic exercise sessions, cognitive exercise sessions, and combined aerobic plus cognitive exercise sessions were conducted using a Motion Fitness Brain Bike^®^ recumbent cycle ergometer. This Motion Fitness Brain Bike^®^ recumbent cycle ergometer is a stationary recumbent bike with a computer mounted such that the participant is able to pedal the cycle ergometer while viewing and interacting with the computer. A mouse was positioned comfortably on the right or left side, depending on the dominant hand of the participant, to allow interaction with the testing and intervention software.

**Table 1:** Group stratification, training schedule, and cognitive training exercises

Group	Participants	Aerobic	Cognition	Flexibility
AER + COG	Cancer	Yes	Yes	Yes
AER	Cancer	Yes	No	Yes
CON	Cancer	No	No	Yes
COG	Cancer	No	Yes	Yes
Week	% HRR	Session	Training exercises	
1	55	1–3	1–5, 1–5, 1–5	
2	55	4–6	1–5, 1–5, 6 (step 10)	
3	55	7–9	6 (step 9–7)	
4	55	10–12	6 (step 6–4)	
5	60	13–15	6 (step 3–1)	
6	60	16–18	1–5, 1–5, 1–5	
7	60	19–21	1–5, 1–5, 7 (step 10)	
8	60	22–24	7 (step 9–7)	
9	65	25–27	7 (step 6–4)	
10	65	28–30	7 (step 3–1)	
11	65	31–33	1–5, 1–5, 1–5	
12	65	34–36	1–5, 1–5, 1–5	

Training exercise numerical values correspond with cognitive training exercises noted in Table 4. The steps indicated during sessions 7–15 and 22–30 reflect a series of tasks within the training exercises of number 6 and number 7 that increase in difficulty as the step number decreases

### Eligibility criteria for participants

Twenty-eight male and female participants undergoing treatment for cancer were eligible to participate in this study. Participant eligibility was initially screened by front office staff prior to his or her arrival at the University of Northern Colorado Cancer Rehabilitation Institute (UNCCRI) for the first physical assessment. Patients were to, initially, be younger than 69.0 years of age, were physically inactive (aerobic exercise < 2.0 times a week), and had not been using any cognitive training software within the 8.0 weeks prior to them coming to UNCCRI for investigators to speak with them further about study participation. Following initial screening and agreement to participate, participants then completed the standard UNCCRI comprehensive physiological assessment detailed in subsequent paragraphs. Following the completion of initial UNCCRI physical assessments, investigators then employed in-person recruitment strategies. It was during these follow-up discussions that investigators would ask potential participants a series of questions that were of greater depth than during the initial round of questions by office staff. Participants were excluded from the study if they reported any of the following: (1) a history of psychiatric disease, (2) any past history of neurological disease, (3) past or present alcohol or substance abuse, (4) difficulty with mobility, (5) auditory dysfunction, or (6) non-corrected visual issues. Qualified participants were then asked for their interest to participate in this study. Those that declined to participate did so verbally, and immediately began the standard UNCCRI exercise programming. The University of Northern Colorado Institutional Review Board approved [573297-2] all procedures, and written informed consent forms were signed by all subjects.

### Setting

Cancer patients were recruited from walk-ins and regional oncologist referrals at UNCCRI located in Greeley, CO, at the University of Northern Colorado. Table 2 indicates group cancer characteristics. Since 1996, UNCCI has been providing individualized, prescriptive exercise to Northern Colorado cancer survivors as part of their recovery from symptoms associated with cancer treatment. Intake of new patients at UNCCRI averages between 3 and 5 patients per week. All training, cognitive and physical assessments, and data collection were conducted at UNCCRI prior to and following the completion of the 36-session intervention.

**Table 2:** Group cancer characteristics

Group	Cancer type	Gender
AER + COG *n =* 9	Breast cancer (4) Ovarian/breast Hodgkin’s lymphoma Non-small cell lung metastasized to the brain Supraglottic/laryngeal Ovarian	Female Female Male Female Male Female
AER *n =* 7	Breast cancer (3) Throat/tongue Anaplastic oligodendroglioma Colon Breast/colon	Female Male Female Female Female
CON *n =* 7	Breast cancer (4) Prostate Ovarian Lymphoma	Female Male Female Male
COG *n =* 5	Breast (3) Lung Multiple myeloma	Female Female Male

### Pre- and post-testing

Following agreement to participate and initial screening, subjects completed a comprehensive physiological assessment [40]. Initial values for blood pressure (BP), heart rate (HR), oxygen saturation (SpO_2_), weight, height, body composition (skinfold measurements), circumference measurements, cardiovascular fitness (VO_2peak_, UNCCRI protocol), balance (Bertec Balance Screener), pulmonary function (spirometry), estimated 1RM (Brzycki equation), muscular endurance (plate-loaded cable-assisted machines, chair squat test, and plank test), handgrip dynamometry, and flexibility measures (modified Sit and Reach and Shoulder Reach Behind Back) were collected as part of the standard UNCCRI physical assessment protocol. Cardiovascular endurance was assessed using the cancer-specific UNCCRI multistage submaximal treadmill protocol [40]. Cognitive function was evaluated prior to and following the completion of each 36-session intervention. The cognitive assessment battery was developed based on a review of studies that have implemented similar methodologies and from recommendations from the faculty in the Department of School Psychology [41]. Cognitive assessments were scheduled aside from physiological assessments, often within the same calendar week, so as to maximize participant effort. Trained doctoral candidates from the Department of School Psychology conducted each assessment of cognitive function. A paper and pen-based battery of well-established cognitive assessment tools were administered in a quiet office in the reception area. All cognitive assessments were in a one-on-one setting (Table 3). Cognitive function was the variable of primary interest.

**Table 3:** Cognitive assessment battery

Cognitive parameters	Test/instrument
General cognitive functioning	• Wechsler Memory Scale, 4th ed. (WMS-IV) • General Cognitive Screener (BCOG)
Processing speed	• Trail Making A (TMT-A)
Working memory, executive function, and attention	• Wechsler Adult Intelligence Scale, 4th ed. (WAIS-IV) • Letter/Number Sequencing, Coding (LNS, CD) • Trail Making B (TMT-B)
Verbal learning and memory	• Wechsler Memory Scale, 4th ed. (WMS-IV) • Logical Memory I & II (LMI, LMII)
Verbal fluidity	• Controlled Oral Word Association Test (COWAT)
Perceptual reasoning	• Wechsler Adult Intelligence Scale, 4th ed. (WAIS-IV) • Block Design (BD)

### Aerobic exercise only intervention (AER)

All exercise sessions were conducted using a Motion Fitness Brain Bike^®^ recumbent cycle ergometer. Sessions were progressive and began at 55.0% of heart rate reserve (HRR, Karvonen method) for weeks 1.0 through 4.0, progressed to 60.0% HRR for weeks 5.0 through 8.0, and finished at 65.0% HRR for weeks 9.0 through 12.0, as detailed in Table 1. Each exercise session consisted of a 5-min warm-up, followed by 30.0 min of exercise at the specified target heart rate. Subjects were encouraged to maintain the target heart rate for the entire session. However, if subjects were unable to maintain the target heart rate, they were allowed to decrease pedaling frequency in order to continue exercising. Following aerobic training participants in this group also completed flexibility training which is detailed below.

### Cognitive training only intervention (COG)

All cognitive training sessions were completed while seated on the Motion Fitness Brain Bike^®^ using NeuroActive^®^ cognitive training software. Cognitive training emphasized activities in working memory, visuo-spatial memory, processing speed, divided attention, selective attention, vigilance, attentional flexibility, useful field of view, verbal processing speed, cognitive control, temporal perception, and arithmetic operations. Each of the cognitive training tasks (parking, driving, smart driving, the policeman, brain twister, the pilot, and the stock exchange) was composed of 5 min of training. Details of each cognitive training component are included in Table 4, and a training schedule for each exercise is provided in Table 1. For the first five sessions, participants completed parking, driving, smart driving, the policeman, and brain twister exercises. On session 6, participants then completed five sessions of the pilot consecutively until session 16.0. During sessions 16–20.0, participants then returned to completing parking, driving, smart driving, the policeman, and brain twister activities. On session 21.0, participants were then required to complete five sessions of the stock exchange consecutively until session 30.0. Finally, during sessions 31–36.0, participants were again required to complete parking, driving, smart driving, the policeman, and brain twister. Following cognitive training, participants in this group also completed flexibility training which is detailed below.

**Table 4:** Cognitive training exercises

Number	Exercise	Trained functions	Description
1	Parking	Working memory Visio-spatial memory	Adaptation and classic visuo-spatial span task
2	Car driving	Processing speed Divided attention Selective Attention Vigilance	Two simultaneous biconditional discrimination (S-R) tasks with a vigilance task
3	Smart driving	Processing speed Selective Attention Attentional Flexibility Useful field of view Divided attention Vigilance	Derived from ACTIVE trial; with UFOV program
4	The policeman	Working memory Verbal processing Speed	Standard *n*-back task with adaptable time limit
5	Brain twister	Processing speed Cognitive control Attentional flexibility	Stroop-like based on cue and response conflict and attentional set-shift paradigm
6	The pilot	Divided attention Temporal perception Arithmetic	Dual monitoring task
7	Stock exchange	Processing speed Divided attention Working memory	Two simultaneous *n*-back tasks: one audio-verbal and the other visuo-spatial

### Flexibility training only intervention (CON)

Flexibility training sessions consisted of 30.0 min of static stretches designed to target major muscle regions throughout the body. Targeted regions included the neck, shoulders/chest, posterior upper arm, upper back, lower back, hips, torso, anterior thigh and hip flexor, posterior thigh, groin, and calf. This flexibility intervention was developed based on detailed flexibility training examples from *the Essentials of Strength and Conditioning* textbook [42].

### Combined aerobic and cognitive training intervention (AER + COG)

Subjects in the combined AER + COG group completed aerobic exercise sessions and cognitive exercise interventions simultaneously (i.e., pedaling at a target heart rate while completing cognitive exercises) as described in the sections above. Subjects in the AER + COG group were encouraged to maintain the target heart rate for the entire session. However, if subjects were unable to maintain the target heart rate, they were allowed to decrease pedaling frequency in order to continue exercising. Following aerobic and cognitive training participants in this group also completed the flexibility training listed above.

### Sample size

This investigation is the result of doctoral work completed by the PI while at the University of Northern Colorado. Sample size was originally intended to exceed 40 participants (G *Power, ver. 3.0.10), yet as the limitations will describe in the upcoming paragraphs, multiple factors influenced the resultant *N* of 28. Throughout the process, the challenge of (1) enrolling willing and eligible participants into the study, (2) frequent attendance to ongoing medical concerns of participants, and (3) the impacts of various occurrences of treatment-related side effects posed the greatest detriments to full completion of 40 participants. As a result, data collection took longer than anticipated. When the time limit for the study was reached, data collection ceased.

### Randomization

Randomization was completed a priori using PROC PLAN (SAS 9.3, Cary, NC), a statistical tool that generates a randomized numerical listing of intervention groups. For example, the AER + COG group was numerically listed as group 1.0, the AER group was listed as group 2.0, the CON group was listed as group 3.0, and the COG group was listed as group 4.0. As participants were pre-screened and qualified for the study, they were then assigned to the next available intervention group on the list. All participants in each group were unaware of the number of different intervention groups. Throughout the study, however, 5.0 subjects presented with specific physician recommendations that required placement into a group other than the random selection, thereby nullifying complete random assignment. The PI enrolled participants and assigned participants based on the next group that appeared on the list.

### Statistical analysis

Pre- to post means, SD, mean differences, and 95.0% confidence intervals for each measure are reported in Tables 5 and 6. With missing data, series mean imputations were applied (SPSS 21, Armonk, NY). Cognitive data (1.3%) that were missing were replaced. Dependent measures *t* tests and pre- to post percentages were calculated to address within-group changes for each dependent variable (SPSS, 21, Armonk, NY). A *p* value of 0.05 was considered statistically significant.

**Table 5:** Summary data: cognitive variables for CON and COG groups

TEST	CON					COG				
	Pre ± SD	Post ± SD	Diff.	95% CI		Pre ± SD	Post ± SD	Diff.	95% CI	
Brief Cog. Screen	54.29 ± 3.20	55.71 ± 4.27	1.43	− 1.39	4.25	53.20 ± 5.31	56.60 ± 1.67	3.40	− 1.91	8.71
Logical Memory I	9.43 ± 2.88	10.71 ± 2.21	1.28	− 0.54	3.11	8.00 ± 3.40	9.60 ± 1.67	1.60	− 0.97	4.17
Logical Memory II	12.14 ± 5.27	11.29 ± 2.36	− 0.85	− 5.70	4.00	9.20 ± 3.70	10.20 ± 2.95	1.00	− 0.76	2.76
Logical Memory %	25.14 ± 3.48	24.87 ± 3.62	− 0.27	− 2.30	1.77	24.40 ± 2.30	24.00 ± 2.34	− 0.40	− 2.28	1.48
Trail Making Test A	29.43 ± 7.96	30.0 ± 11.10	0.57	− 4.66	5.80	29.20 ± 11.00	31.70 ± 9.92	2.49	− 6.61	11.60
Trail Making Test B	74.71 ± 22.51	72.42 ± 46.84	− 2.29	− 31.03	26.47	92.60 ± 34.65	69.00 ± 20.50	− 23.62	− 64.91	17.68
Block Design	10.43 ± 2.51	11.00 ± 1.91	0.57	− 1.87	3.01	7.80 ± 1.64	9.00 ± 1.41	1.20	− 0.42	2.82
Letter Num. Seq.	10.60 ± 2.22	10.76 ± 2.86	0.16	− 1.08	1.40	9.60 ± 0.90	9.60 ± 0.55	0.00	− 1.52	1.52
Coding	10.71 ± 1.60	11.43 ± 3.31	0.71	− 1.53	2.96	12.00 ± 1.58	11.80 ± 1.92	− 0.20	− 1.81	1.42
COWAT-G	0.06 ± 0.83	0.51 ± 0.84*	0.45	0.07	0.83	− 0.14 ± 0.78	0.08 ± 0.66	0.22	− 0.37	0.82
COWAT-A	0.15 ± 0.86	0.63 ± 0.85*	0.48	0.12	0.83	− 0.04 ± 0.75	0.18 ± 0.67	0.22	− 0.37	0.82
COWAT-ED	− 0.22 ± 0.91	0.23 ± 1.00*	0.45	0.07	0.83	− 0.62 ± 0.63	− 0.25 ± 0.60	0.37	− 0.27	1.01
VO_2peak_	16.93 ± 8.25	19.59 ± 7.59*	2.66	0.58	4.73	20.08 ± 4.88	22.50 ± 5.21	2.42	− 2.89	7.73

Confidence intervals (CI) are presented as intervals of mean difference between pre- and post-values

*WMS IV* Weschler Memory Scale (4th Ed.) (*BCOG* Brief Cognitive Screener; *LMI & LMII* logical memory delayed recall (DR) or cumulative percentage (CP)); *TMT A or B* Trail Making Test version A or B, *WAIS IV* Weschler Adult Intelligence Scale (4th Ed.) (*BD* block design, *LNS* letter number sequence, *CD* coding); *COWAT* Controlled Oral Word Association Test (*Z Z*-score, *G* gender, *A* age, *ED* education). *VO*_*2peak*_ = the highest rate of oxygen consumed measured regardless of reaching VO_2_ plateau. Diff = difference between pre- and post-values. The asterisk (*) denotes results were significant (*p* < 0.05)

**Table 6:** Summary data: cognitive variables for AER and AER + COG groups

TEST	AER					AER + COG				
	Pre ± SD	Post ± SD	Diff.	95% CI		Pre ± SD	Post ± SD	Diff.	95% CI	
Brief Cog. Screen	54.00 ± 5.26	52.71 ± 4.54	− 1.29	− 7.82	5.25	54.56 ± 3.64	54.67 ± 3.53	0.11	− 2.65	2.87
Logical Memory I	7.00 ± 1.73	9.29 ± 2.29*	2.29	0.90	3.67	10.33 ± 3.00	10.33 ± 2.00	0.00	− 1.63	1.63
Logical Memory II	7.86 ± 3.13	10.00 ± 2.31*	2.14	0.34	3.95	11.44 ± 2.74	12.22 ± 2.17	0.78	− 0.84	2.40
Logical Memory %	24.00 ± 2.94	24.28 ± 3.15	0.29	− 1.96	2.53	26.89 ± 2.47	26.22 ± 1.20	− 0.67	− 2.63	1.30
Trail Making Test A	36.86 ± 7.60	36.43 ± 14.19	− 0.43	− 12.15	11.30	60.44 ± 85.02	30.67 ± 10.00	− 29.78	− 90.02	30.47
Trail Making Test B	87.29 ± 9.79	98.14 ± 34.19	10.86	− 16.89	38.60	69.44 ± 20.34	72.22 ± 29.75	2.78	− 9.62	15.18
Block Design	9.00 ± 1.83	10.71 ± 1.11*	1.71	0.23	3.20	9.00 ± 2.96	9.89 ± 2.89	0.89	− 0.72	2.50
Letter Num. Seq.	8.14 ± 0.70	9.14 ± 0.90*	1.00	0.24	1.76	11.56 ± 3.43	11.44 ± 2.88	− 0.12	− 2.16	1.94
Coding	9.14 ± 1.46	10.29 ± 1.38	1.14	− 0.81	3.10	11.78 ± 3.87	11.78 ± 4.06	0.00	− 0.54	0.54
COWAT-G	− 0.54 ± 0.31	− 0.63 ± 1.10	− 0.08	− 0.93	0.77	− 0.04 ± 1.60	0.23 ± 1.80	0.27	− 0.26	0.81
COWAT-A	− 0.46 ± 0.37	− 0.14 ± 0.61	0.31	− 0.14	0.76	0.10 ± 1.57	0.38 ± 1.78	0.28	− 0.25	0.81
COWAT-ED	− 0.80 ± 0.56	− 0.45 ± 0.81	0.35	− 0.20	0.90	− 0.20 ± 1.52	− .17 ± 1.75	0.03	− 0.66	0.72
VO_2peak_	18.67 ± 5.43	22.40 ± 8.00	3.72	− 2.14	9.60	21.07 ± 8.35	21.62 ± 8.48	0.56	− 2.13	3.25

Confidence intervals (CI) are presented as intervals of mean difference between pre- and post-values

*WMS IV* Weschler Memory Scale (4th Ed.) (*BCOG* Brief Cognitive Screener, *LMI & LMII* logical memory delayed recall (DR) or cumulative percentage (CP)); *TMT A or B* Trail Making Test version A or B, *WAIS IV* Weschler Adult Intelligence Scale (4th Ed.) (*BD* block design, *LNS* letter number sequence, *CD* coding), *COWAT* Controlled Oral Word Association Test (*Z Z*-score, *G* gender, *A* age, *ED* education). VO_2peak_ = the highest rate of oxygen consumed measured regardless of reaching VO_2_ plateau. Diff. = difference between pre- and post-values. The asterisk (*) denotes results were significant (*p* < 0.05)

## Results

Twenty-eight cancer patients (57.9 ± 8.0 years; 6 males, 22 females) participated in the study. Subjects in the CON group showed some pre- to post improvements in Verbal Fluidity scores only. It was hypothesized that cognitive training alone and aerobic training alone would each, independently, improve cognitive function. While subjects in the AER group did show improvements, the COG group showed no improvements in cognitive function. The AER group showed pre- to post improvements in logical memory scores (pre- to post mean difference [2.3], 95.0% CI [0.9, 3.7], percentage change [32.7%]), delayed recall scores (pre- to post mean difference [2.1], 95.0% CI [0.3, 3.9], percentage change [27.2%]), block design scores (pre- to post mean difference [1.7], 95.0% CI [0.2, 3.2], percentage change [19.0%]), and letter-number sequencing scores (pre- to post mean difference [1.0], 95.0% CI [0.2, 1.8], percentage change [12.3%]).

## Discussion

The development of holistic, scientifically based strategies for treatment and rehabilitation are necessary to address the negative aspects of cancer. Current methods of prevention, detection, education, and treatment, along with individualized exercise-based cancer rehabilitation programs, may play a significant role in this process. CRCI is a disruptive and frustrating phenomenon among cancer survivors that can significantly impact quality of life. Although sample sizes were small and data were non-normal, this project addressed factors that potentially aid in reducing or attenuating CRCI and provides a workable model that may be modified and implemented again in future randomized controlled trials or at large-scale multi-site cancer centers.

Subjects in the CON group showed pre- to post improvements in COWAT (gender, age) *Z*-scores but not with any other test. In a more recent study evaluating the effects of Qigong as a component of a cancer rehabilitation program, results indicated that flexibility training may be an effective rehabilitative modality [43]. Specifically, Qigong was frequently associated with increases in QOL and spiritual well-being and decreases in anxiety, depression, and fatigue. However, empirical evidence regarding specific physiological changes that may account for these beneficial effects that occur with flexibility training is lacking. Likewise, few studies have evaluated the effects of static stretching, yoga, or Qigong on cognitive function, and the studies that have been completed utilized cognitive assessment batteries that differed significantly from the present study; no studies exist which investigate cancer survivors. However, an investigation of the effects of a 6-month yoga-based intervention on measures of cognitive function among apparently healthy adults over 60.0 years of age did report improvements. Significant increases were observed among delayed recall of visual, working, and verbal memory; executive function; working memory and attention; processing speed; and verbal fluency post intervention [44]. No other measure of cognitive function significantly increased for the CON group except the abovementioned verbal fluidity scores, which only partially substantiate these results. It may be a possibility that the social interaction and one-on-one personal training of our program played a role in increased verbal fluency.

Observations from the COG group in this study are not in agreement with other studies that have shown increases in aspects of cognitive function as a result of cognitive training [45, 46]. To possibly explain this incongruity, we noted the following observations: (a) the level of difficulty of the NeuroActive^®^ intervention was consistently high and may not be entirely appropriate for those who are undergoing treatment for cancer, (b) the type of games may not have been engaging or stimulating enough to encourage full cognitive engagement from the participants, or (c) the software itself failed to accurately train cognitive processes as its designers suggested it would. Many study participants stated on multiple occasions that the tasks were difficult and often left them feeling overwhelmed and frustrated.

In a more recent study, apparently healthy males that exercised at a moderate intensity (65.0% HRR, 20.0 min) significantly increased cognitive accuracy and speed (Stroop test) when compared to 10.0 or 45.0 min of cycling, suggesting a dose-response relationship between aerobic exercise and cognitive function [47]. In the current study, the AER group showed the greatest improvement in pre- to post cognitive changes with significant increases in WMS-LMI scaled scores (32.8%) and WMS-LMII DR scaled scores (26.6%). A study evaluating the effects of 7 weeks of aerobic training (50–75.0% HRR) on brain volumetric changes in apparently healthy older adults revealed significant increases in memory and hippocampal volumes [48]. Aerobic training in this study impacted aspects of memory, but for cancer survivors, intensities may need modification to account for the added physical and cognitive demand of cancer treatment. WAIS-BD scaled scores (18.9%) and WAIS LNS scaled scores (12.3%) also significantly increased suggesting that perceptions and processing of the test visual components increased potentially as a result of aerobic exercise. Participants in the AER group showed more broad and consistent improvements in cognitive function than other study groups. Cognitive improvements as a function of moderate intensity (65.0% HRR) aerobic exercise have been suggested to follow an inverted U paradigm such that an optimal intensity and time may yield the greatest amount of exercise-related cognitive changes, at least among apparently healthy males and sedentary male and female adults older than 65 years [49, 50].

The virtual absence of any beneficial effects observed in the AER + COG group may be a direct result of the level of difficulty reported among patients who completed the combined training protocol. Thirty minutes of aerobic cycling was attempted at 55.0, 60.0, and 65.0% (HRR) in this study; however, maintaining intensities was often difficult to accomplish because individual functional capacity varied daily. Although results suggest that aerobic training alone produced significant pre- to post increases in measures of cognitive function, perhaps between 40.0 and 55.0% HRR may more appropriately reflect tolerable physiological levels of exertion for this population. The lack of improvements in cognitive function observed in the COG group supports this. In addition, it appears as though combining aerobic exercise with cognitive exercise exacerbated the level of difficulty. This suggests that a neurological conflict may have been occurring such that the ability of the brain to appropriate the amount of processing capacity between two difficult tasks was challenged. Patients were extremely frustrated with the difficulty of the combined training, often indicating that the level of concentration required to perform both physical and cognitive exercises was overwhelming.

To our knowledge, there have been no studies examining the effects of a combined aerobic and cognitive training intervention on cognitive function in cancer survivors. It is imperative that combined cognitive and aerobic training at a moderate intensity not be overlooked with regard to CRCI reduction. We do not believe that the lack of a response in this group is a result of combining exercise training with cognitive training per se. Rather; we believe that the lack of a response was the result of the level of difficulty associated with each intervention. It is possible that the measureable benefits would have been realized had the exercise been at a lower intensity, if the cognitive intervention would have been less taxing, or both. In addition, it is possible that combined training may be too demanding for some individuals to do well at both cognitive and aerobic training simultaneously. An alternative may be to complete both aerobic and cognitive training, but not simultaneously.

For the exercise treatment group, 30.0 min of aerobic cycling at a self-reported moderate intensity was observed to produce increases in cognitive function. For cancer survivors in a rehabilitation setting, these results further reinforce the importance of aerobic training, particularly those programs following an intensity-based regimen. In cases where side effects of treatment became overwhelming (e.g., fatigue, depression, nausea, diarrhea), the adaptability of the training increased participant tolerance and completion. When such events occurred, a reduction in intensity may be appropriately substituted with rating of perceived exertion (RPE) in order to continue training. While flexibility training was used as an ethically appropriate intervention for the control group that would not force subjects to be on a “wait list,” unexpected improvements in verbal fluidity were observed. Flexibility training should be further investigated to determine if this is in fact a viable intervention to improve CRCI and, if so, identify possible mechanisms of action. If this is indeed the case, flexibility interventions may be incorporated to a greater extent in cancer rehabilitation, which may be considered a more ethically appropriate control group for studies conducted in cancer survivors as opposed to the “wait list” control group.

Cognitive training alone may be an effective strategy in reducing CRCI. Other cognitive-based interventions have shown improvements in cognitive function across many different populations, but a few have aimed to address CRCI in cancer survivors. However, our experience suggests that the NeuroActive^®^ software may not be appropriate for this population, considering its level of difficulty and the low patient-reported satisfaction. Utilization of a cognitive intervention more appropriately targeting cancer survivor populations may have resulted in greater improvements in cognitive function in both the COG and AER + COG groups.

## Conclusions

The investigators of this pilot study aimed to address a debilitating cancer treatment-related side effect. We attempted to evaluate how two independent interventions may be utilized to reduce CRCI and thereby increase quality of life in cancer survivors. These data provide a framework for future studies aiming to utilize aerobic exercise in the attenuation of CRCI. More research must be done to fully examine the effects of combined cognitive and aerobic training on cognitive function, but until then, aerobic exercise may be as an easily implementable modality to address CRCI in cancer survivors.

### Limitations

The complexities of cancer were the greatest limitations experienced during this study. When patients are referred to UNCCRI, their presentation of physiological and psychological symptoms may vastly differ from one another along the cancer continuum. This may include different cancer stages, types of cancer, types of treatment, combinations of types of treatment, and individualized physiological and psychological responses to the treatment itself. One prominent example was a woman who was pre-screened and met qualification standards for this study. She was undergoing treatment for stage III brain cancer and would often have moments where she would randomly cease talking, slow or stop cycling, and appear to be awake, but incoherent. It was determined that because of her type of cancer, treatment, and being on frequently oscillating dosages of gabapentin, this was something that needed to be addressed on a daily basis but did not fit the criteria for exclusion. Since many were undergoing treatment, dosages and regimens often changed, and because oftentimes participants did not feel well during physical or cognitive training, aerobic training had to be reduced to a comparable RPE so training could be completed. Medical emergencies, cancer recurrence, inclement weather, holidays, or last minute appointment changes with physicians were also significant factors that affected data collection. This study provides an important starting point for future studies evaluating the impacts of various modalities on CRCI. However, with everything in consideration, the results of this study should be interpreted with caution.

## Abbreviations

AER + COG: Aerobic and cognitive training group
AER: Aerobic training only group
COG: Cognitive training only group
CON: Flexibility training only group
COWAT-A: Controlled Oral Word Association Test-age
COWAT-ED: Controlled Oral Word Association Test-education
COWAT-G: Controlled Oral Word Association Test-gender
CRCI: Cancer- or chemotherapy-related cognitive impairment
HRR: Heart rate reserve
RPE: Rating of perceived exertion
WAIS-BD: Weschler Adult Intelligence Scale block design
WAIS-CD: Weschler Adult Intelligence Scale coding
WAIS-LNS: Weschler Adult Intelligence Scale letter number sequence
WMS-LMI: Weschler Memory Scale logical memory I
WMS-LMII DR: Weschler Memory Scale logical memory II-delayed recall
